# How Surface Properties of Silica Nanoparticles Influence Structural, Microstructural and Biological Properties of Polymer Nanocomposites

**DOI:** 10.3390/ma14040843

**Published:** 2021-02-10

**Authors:** Łukasz Zych, Anna Maria Osyczka, Agnieszka Łacz, Agnieszka Różycka, Wiktor Niemiec, Alicja Rapacz-Kmita, Ewa Dzierzkowska, Ewa Stodolak-Zych

**Affiliations:** 1 Department of Ceramics and Refractories, AGH University of Science and Technology, 30-059 Krakow, Poland; lzych@agh.edu.pl (Ł.Z.); kmita@agh.edu.pl (A.R.-K.); 2 Department of Cell Biology and Imaging, Institute of Zoology and Biomedical Research, Jagiellonian University, 30-387 Krakow, Poland; a.m.osyczka@uj.edu.pl; 3 Department of Inorganic Chemistry, AGH University of Science and Technology, 30-059 Krakow, Poland; alacz@agh.edu.pl; 4 Department of Building Materials Technology, AGH University of Science and Technology, 30-059 Krakow, Poland; ar@agh.edu.pl; 5 Department of Silicates and Macromolecular Compounds, AGH University of Science and Technology, 30-059 Krakow, Poland; wniemiec@agh.edu.pl; 6 Department of Biomaterials and Composites, AGH University of Science and Technology, 30-059 Krakow, Poland; dzierzkowska@agh.edu.pl

**Keywords:** nanoparticles, silica, nanocomposite, bioactivity, bone substitute, regenerative medicine

## Abstract

The aim of this work was to study effect of the type of silica nanoparticles on the properties of nanocomposites for application in the guided bone regeneration (GBR). Two types of nanometric silica particles with different size, morphology and specific surface area (SSA) i.e., high specific surface silica (hss-SiO_2_) and low specific surface silica (lss-SiO_2_), were used as nano-fillers for a resorbable polymer matrix: poly(L-lactide-co-D,L-lactide), called PLDLA. It was shown that higher surface specific area and morphology (including pore size distribution) recorded for hss-SiO_2_ influences chemical activity of the nanoparticle; in addition, hydroxyl groups appeared on the surface. The nanoparticle with 10 times lower specific surface area (lss-SiO_2_) characterized lower chemical action. In addition, a lack of hydroxyl groups on the surface obstructed apatite nucleation (reduced zeta potential in comparison to hss-SiO_2_), where an apatite layer appeared already after 48 h of incubation in the simulated body fluid (SBF), and no significant changes in crystallinity of PLDLA/lss-SiO_2_ nanocomposite material in comparison to neat PLDLA foil were observed. The presence and type of inorganic particles in the PLDLA matrix influenced various physicochemical properties such as the wettability, and the roughness parameter note for PLDLA/lss-SiO_2_ increased. The results of biological investigation show that the bioactive nanocomposites with hss-SiO_2_ may stimulate osteoblast and fibroblast cells’proliferation and secretion of collagen type I. Additionally, both nanocomposites with the nanometric silica inducted differentiation of mesenchymal cells into osteoblasts at a proliferation stage in in vitro conditions. A higher concentration of alkaline phosphatase (ALP) was observed on the material modified with hss-SiO_2_ silica.

## 1. Introduction

Surgical procedures related to bone grafting are about 2 million orthopedic surgeries per year worldwide annually [1]. This demand for traditional solutions has led to a situation in which the Tissue Banks lack natural substitutes filling the defect and support rapid regeneration of bone tissue (the so-called gold standard).

Numerous scientific studies indicate that synthetic bone substitutes (BGSs) should meet several features ensuring maximum structural and morphological fit to natural bone (structural and microstructural biomimetics) [2,3,4].

The ideal bone substitute should have the ability to allow space (scaffold) for the incoming cells and, at the same time, facilitate tissue reconstruction including neovascularization or bone canals [5]. Only the scaffold providing osteointegration into the tissue, i.e., providing conditions for cell adhesion and proliferation and giving a signal for cell differentiation, is currently the direction identified with the solution of many clinical problems [6,7,8,9]. Such problems include, for example, ceramic and synthetic bone substitutes based on hydroxyapatite (HAp), tricalcium phosphates (TCP), and bio glasses (BG) [10,11,12]. Completely resorbable porous ceramic materials mechanically based on TCP or BG provide osteoinductivity and osteoconductivity but do not meet the mechanical requirements [13,14]. Low mechanical strength (less than the cortical bone), brittleness, and low handiness (difficulty to cut under operating conditions) are a problem in clinical conditions. However, modifications in the chemical structure of apatite (Si, Sr, Fe, and Mg ion substitution) and bioglass (addition of MgO, SrO, or ZnO oxides) allow to achieve better structural mimetics of the substitute, but do not significantly improve its mechanical properties while maintaining high bioactivity [15,16,17,18,19].

The second material approach is the use of composites and nanocomposites based on resorbable polymers inducing the right cellular response. Polylactides are commonly praised for their excellent mechanical properties (e.g., high modulus and yield strength) and possibility to control durability of an implant in in vitro conditions (e.g.,; by different isomeric L to DL ratio). PLDLA has a structure that combines the best characteristics of poly (L-lactic acid) and poly (D-lactic acid), i.e., the mechanical properties of the first and the shorter degradation time of the second [1,4]. Among bioresorbable polymer materials, poly-L/DL-lactide (PLDLA) has FDA approval, which guarantees its application as an implant in orthopedic medicine. Our study confirms that the presence of inorganic nanoparticles (i.e.,; SiO_2_) stimulating bone cells growth on resorbable implant based on PLDLA is an effective method for initiating the regeneration process.

Polymer–ceramic nanocomposites may contribute to the production of biocompatible implant materials with adequate strength and are capable of stimulating the reaction of repair cells and proliferation of bone cells [20,21,22,23]. This task may be accomplished by altering a surface energy and/or surface topography of the nanocomposite by the addition of a specific amount of bioactive nanoparticles such as silica [24,25,26]. Nanocomposites filled with bioactive ceramic nanoparticles such as nanometric tricalcium phosphate (TCP) or hydroxyapatite (HAp) are examples of such biomimetic materials. The main task of a bioactive nanofiller is to induce a specific biological reaction leading to a formation of a bond between natural bone and an implant material [21,23,27]. Because of their high specific surface area, which is inversely proportional to the particles size, the nanoparticles may have a strong impact on the surrounding material. Nanoparticles, as it is known from the literature, are characterized by a different ratio between mass and area than conventional particles of micrometric size.

Silicon dioxide (SiO_2_) or silica is a ceramic additive, which was successfully applied in implant materials for bone surgery. Up to now, it has mainly been deposited as layers or applied in the form of particles modifying the implant’s surface [28,29,30]. Silica plays the crucial role in the bio-mineralisation process by enhancing bioactivity of a material due to the presence of silanol groups (SiOH-) at its surface, which favours the formation of hydroxyapatite in in vivo conditions [31,32,33]. Bioactivity of micrometric silica particles depends on its surface charge, composition, structure, and morphology. During their immersion in the simulated body fluid (SBF), a significant amount of silanol groups appeared, which then led to the formation of silica gel and consequently to apatite layer. The phenomenon of apatite formation on silica was explained by numerous studies [34,35]. According to them, adsorption of calcium ions, which was stronger than adsorption of phosphate ions, is the initial step of apatite nucleation. This phenomenon strongly depends on the pH of the solution as well as on zeta potential of the silica particles [35,36,37].

Nanometric silica having a high specific surface and thus a high amount of exposed silanol groups seems to be an interesting osteoconductive filler in nanocomposites destined for bone implants [27,34]. The incorporation of silica nanoparticles into the polymer matrix stimulated osteoblast-like cells interaction with natural tissue after contact with the material’s surface i.e.,; cells viability increased since Si (at critical concentrations) is able to stimulate proliferation of MG-63 cells [24,38]. Si also can be involved in bone formation and mineralization [39], whereas orthosilicate acid (Si(OH)_4_) at a physiological concentration of 10 μmol was shown to stimulate the formation of collagen type I in human osteoblast cells (HOC) and osteoblastic differentiation [40].

The experimental approach in the topic of bone tissue regeneration with bone substitutes consists in the preparation of a polymer substrate on which the patient’s own cells are applied. In some cases, they have been used as bioactive carriers providing local osteoinductive genes—starting the bone regeneration process. For this purpose, mesenchymal stem cells, which are multi-potential cells capable of effective use of osteogenic potential, are widely used. In our work, we show that a necessary condition to stimulate the regeneration process with the use of nanocomposite materials based on PLDLA by mesenchymal stem cells is the presence of an appropriate active nanoparticle. The comparison of silica nanoparticles shows that not all of them have the same chemical potential that can have a real impact on the processes related to the metabolic activation of cells i.e.,; proliferation or secretion. The proposed research methodology used for the nanoparticles allows to select the nanoparticle with the highest bioactive potential. It could be used instead of performing a series of tedious studies on the preparation of a homogeneous nanocomposite that could have bioactive properties.

## 2. Materials and Methods

Poly(L-lactide-co-D,L-lactide) polymer (Boehringer Ingelheim, Ingelheim am Rhein, Germany) was used as a matrix material. Molecular weight of the polymer was 200 kDa, and L-lactide to D,L-lactide ratio was 80:20. The polymer matrix was modified using nanometric powders of both low specific surface area silica—lss-SiO_2_ (Arco S.A., Poznan, Poland), and high specific surface area silica—hss-SiO_2_ (Sigma Aldrich Com, Munich, Germany).

Polymer–ceramic nanocomposite membrane preparation: polymer-ceramic nanocomposite materials in the form of thin foils were produced using a casting method. The polymer was dissolved in dichloromethane (CH_2_Cl_2_, POCh S.A.) (1:10 wt. ratio) and then 0.5 wt.% of a given silica powder dispersed in CH_2_Cl_2_ was introduced and ultrasonicated for 5 min. After mechanical stirring of the polymer solution and the silica dispersion, the polymer mixture was cast on a Petri dish and then the solvent was evaporated at room temperature (6 h) using a dryer (80 °C/24 h).

The silica powders were analysed in terms of particle size distribution (DLS method, Zetasizer Nano-ZS, Malvern Ins., Worcestershire, United Kingdom), particle morphology (TEM, JEOL—JEM1011, Freising, Germany), specific surface area (BET), and porosity (nitrogen adsorption at 77K, BJH method; Nova 1200e, Quantachrome Inc., Boynton Beach, FL, USA). The presence of surface groups was investigated using FT-IR (Bio-Rad Excalibur spectrometer, Bio-Rad Polska Sp. z.o.o., Warszawa, Polska). For the infrared analysis, powder samples were prepared in the form of potassium bromide (KBr) pellets (transmission mode). For better analysis of the nanoparticles surface, the Diffuse Reflection mode (DRIFTs) was used. The silica powders were diluted in a nonabsorbent matrix (KBr). Additionally, zeta potential changes of the silica powders immersed in SBF with time were determined by Laser-Doppler Velocimetry Electrophoresis method (Zetasizer Nano-ZS, Malvern Ins., Worcestershire, UK).

In order to verify if the produced materials have nanocomposite character, they were subjected to thermal analysis using differential scanning calorimetry in an inert gas atmosphere (helium) at temperatures from 50 °C to 300 °C, with a heating rate of 10 °C/min (DSC 2010, TA Instruments).

The effect of SiO_2_ nanoparticles on material topography was determined using an atomic force microscope (AFM, Bruker MultiMode V microscope, Digital Inst., Burlington, MA, USA). The contact mode was used, which determined the most important topography parameters i.e., Ra (mean surface roughness) and RMS (effective roughness) over a 100 × 100 µm test area. The sensitivity of the measurement system was less than 100 Å. Wettability of the surface with polar and non-polar liquid was measured using a goniometer (DSA 25, Kruss, Hamburg, Germany) by the direct measurement method. The tests were carried out at atmospheric conditions using high purity water (UHQ, PURE Lab, Vivendi water, Poznan, Poland) as the liquid for wettability determination and diiodomethane as the non-polar liquid. The surface free energy was determined using the Owens–Wendt method.

A bioactivity test was performed by incubation of all materials in simulated body fluid (SBF), and the biological response was assessed based on the interaction with fibroblasts (HS-5, ATCC) and osteoblasts of rats (I Local Ethics Committee in Krakow, Poland No. 25/2007). SBF is an artificial body fluid with pH and ion concentration similar to human body fluid, but free from proteins and cells, and its chemical composition is presented in Table 1. The incubation enabled to create in vitro conditions, facilitating the formation of an apatite layer on the material’s surface. The samples were incubated at 37 °C for 3 days. The microstructure of the samples after 3 days of SBF incubation was examined using scanning electron microscope (JMS-5400, JEOL, Osaka, Japan).

Fibroblasts (HS-5, ATCC CRL-11882) and osteoblasts (MG-63, ATCC CRL-11372) of 2 × 10^−4^ concentration were cultured on the investigated materials inside 12 h`ole culture plates. For fibroblast cells, RPMI culture medium (Sigma Aldrich Com, Munich, Germany) was applied enriched in 15% of fetal calf serum (FCS). Temperature of the culture was 37 °C. In the case of osteoblast-like cells, a 1:1 mixture of F12 HAM nutrient medium and Dulbecco modification of Eagle medium (DME F12, Sigma Aldrich Com, Munich, Germany) was applied without phenol red with the addition of 10% of fetal calf serum. The culture temperature was 34 °C. Viability of cells was determined using CellTiter 96 AQueous One Solution Cell Proliferation Assay (MTS, Promega, Madison, WI, USA). Collagen type-I concentration was determined using Collagen Type I ELISA test using Bioproducs (Lussane, Switzerland) antibodies and standards. The statistical analysis was based on Fisher’s exact test and Student’s *t*-test.

Trabecular bone fragments were obtained from the bone of rats (I Local Ethics Committee in Krakow, Poland No. 25/2007). Cell isolation preparation was based on the protocol first described by Robey and Termine [41,42]. A fragment of rat spongy bone was collected and minced [ethics committee approval]. Tissue homogenization was carried out by shredding with surgical scissors in DMEM/F-12K (Sigma Aldrich Com, Munich, Germany) enriched with antibiotics (50 IU penicillin/mL, 50 mg streptomycin/mL, Sigma Aldrich Com, Munich, Germany). In the next step, the biological material was incubated in immersion medium under conditions simulating a living organism: 37 °C/3–4 h in an air atmosphere with 5% CO_2_ until the cellular material on the bone surface disappeared. The immersion medium consisted of DMEM/F-12K, 2mM L-glutamine, 50 lg/mL ascorbate, 256 U/mL collagenase type XI, and antibiotics. The minced bone fragments were then rinsed with 0.9% sodium chloride (Baxter). The cultures were prepared by incubating the biological material in medium containing calcium-free DMEM/F12-K supplemented with 10% fetal bovine serum (FBS), 2 mM L-glutamine, 50 lg/mL ascorbate, and antibiotics. The cultures were kept at 37 °C/3–4 days in an air atmosphere with 5% CO_2_. The confluent cell layer was trypsinized (0.25% trypsin containing 1 mM EDTA, Sigma Aldrich Com, Munich, Germany) and counted in a hemocytometer.

The cell viability on the surface of the pure polymer and nanocomposite materials was performed using prime culture of mesenchymal cells (MSCs). Viability of cells was determined using CellTiter 96 AQueous One Solution Cell Proliferation Assay (MTS, Promega GmbH, GmbH, Madison, WI, USA). The ALP activity test was performed using a commercial ELISA substrate kit (Life Technologies Polska Sp. z. o. o., Warsaw, Poland). ALP activity was tested at three time intervals: 7, 14, and 21 days of the cell culture. After adequate cell culture time, the nanocomposite films were washed with PBS and then 400 μL of p-nitrophenyl phosphate was added to each well; after 30 min of incubation, 200 μL of 2 M NaOH was added to inhibit the reaction (hydrolysis of p-nitrophenyl phosphate to p-nitrophenol and phosphate, causes color change). The absorbances of the obtained solution at 405 nm were read using a microplate reader (GloMax^®^ Discover Microplate reader, Promega GmbH, Madison, WI, USA).

## 3. Results

One of the most important factors effecting properties of nanocomposite materials is the characteristics of applied additives (fillers). Since in both nanocomposite materials the amount of silica was the same (0.5% w/w) and they were prepared in the same manner, the only reason for changes in their behavior could be differences in the filler characteristics.

**Figure 1:**
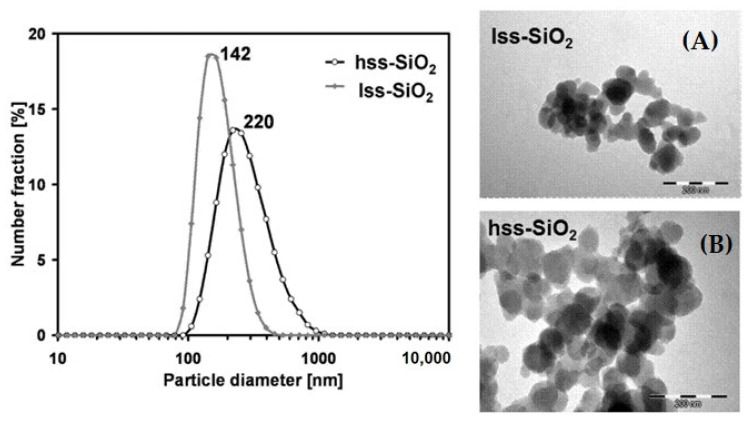
Particle size distribution of silica powders determined by DLS method (water, pH = 2) and TEM morphology of: lss-SiO_2_ (**A**) and hss-SiO_2_ (**B**) powders.

**Nanoparticles characteristics:** Particle size distribution in the case of hss-SiO_2_ powder was broad with a modal value of 220 nm and narrower in the case of lss-SiO_2_ with a modal value around 140 nm (Table 2). Particle size distributions were determined by DLS method in diluted, well dispersed (pH = 2) water suspensions, and the results may be treated as the limit distribution, which can be achieved in conditions of very effective dispersion of the powders (Figure 1).

Probably in the case of actual silica dispersions used for the composites’ production, the agglomerate size was larger. Mean size of the primary particles estimated on the basis of TEM microphotographs (Figure 1) was c.a. 50 and 60 nm in the case of lss-SiO_2_ and hss-SiO_2_, respectively. The comparison between the primary particles size (TEM) and agglomerates size (DLS) suggested that the applied dispersion method did not sufficiently break the agglomerates, because there was no evidence of the primary particles in the suspension. TEM microphotographs suggested that wide contacts between the primary particles were formed, which means that breaking them with the use of sole ultrasounds might be very difficult or even impossible.

**Figure 2:**
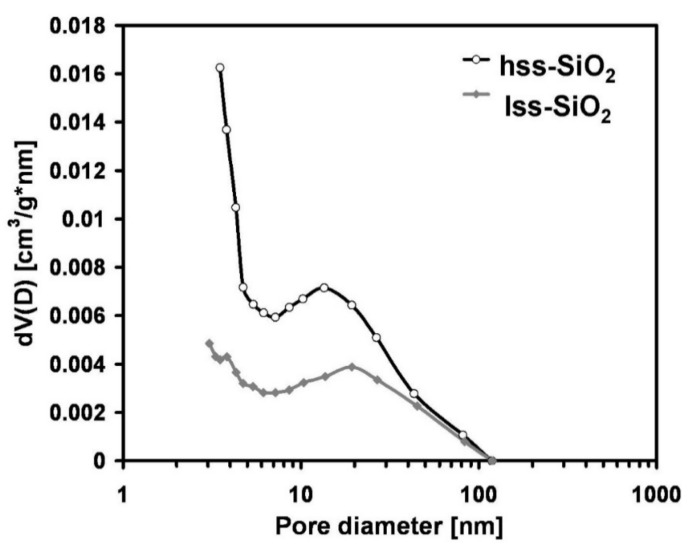
Pore size distribution of the silica powders determined from the desorption branch of nitrogen adsorption/desorption isotherms using BJH method (temperature 77 K).

Despite a rather similar particle size, both powders revealed quite different specific surface areas (Table 2), which were 582.8 m^2^/g and 65.8 m^2^/g for hss-SiO_2_ and lss-SiO_2_, respectively. The high specific surface area of hss-SiO_2_ was related to the presence of a relatively high amount of micropores i.e., pores of sizes smaller than 2 nm (according to IUPAC definition) (Figure 2). Due to technical limitations of the apparatus used, it was impossible to determine pores smaller than 2 nm, but the pore size distribution curves may suggest that such pores existed in both silica powders. In the case of lss-SiO_2,_ the number of such pores was smaller, and the porosity mainly consisted of mesopores i.e., pores of diameter between 2 and 100 nm. Such pores existed in both powders and most probably were related to intra-agglomerate porosity.

**Figure 3:**
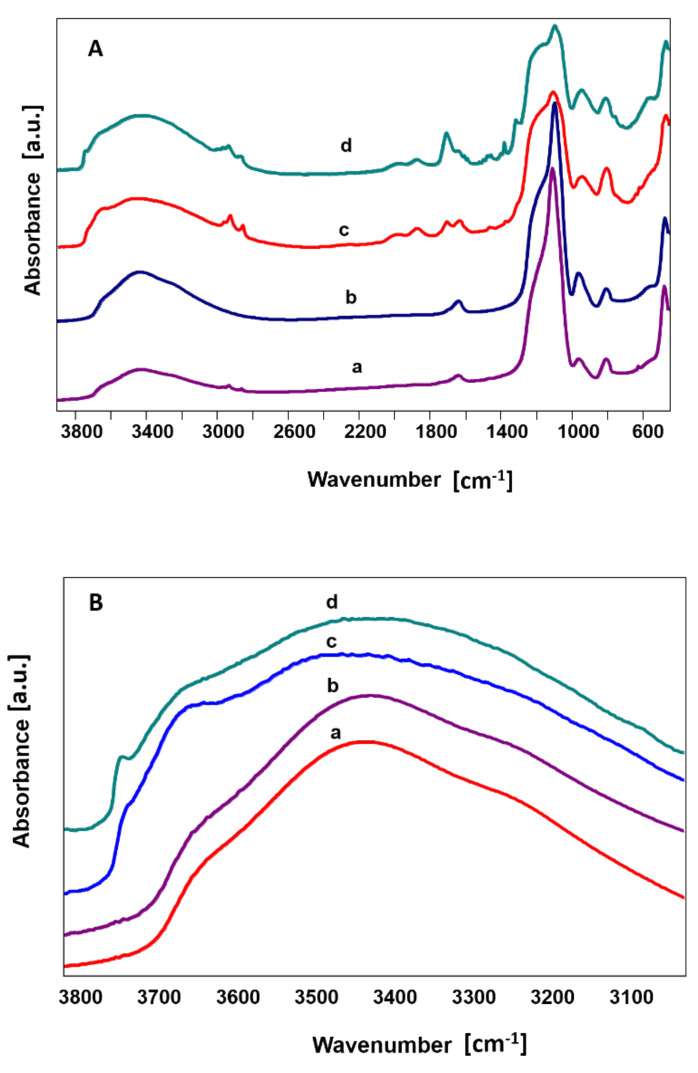
FTIR spectra of silica nanoparticles: lss-SiO_2_ (a,c) and hss-SiO_2_ (b,d) in all spectrum 580–3800 cm^−1^ (**A**). FTIR spectra in transmission mode (a,b) and in DRIFTs mode (c,d) in the range 3000–3800 cm^−1^ (**B**).

FTIR spectra of the silica nanoparticles showed subtle differences between the powders. Conventional transmission mode indicated typical bands belonging to Si-O-Si bending vibrations at 470 cm^−1^ and 810 cm^−1^ and Si-O groups in the range of 1100 cm^−1^ belonging to stretching vibrations. Bands at 960 cm^−1^ are characteristic for Si-OH stretching vibration [43,44]. The bands in the region 3650 to 3750 cm^−1^ belonged to hydrogen bonding (Figure 3A, curves: a,b). These results were confirmed using DRIFTs technique (Figure 3A, curves c,d). Stronger bands in 1680 cm^−1^ confirmed hydrogen-bonded water present on the silica surface. The precise analysis of the FTIR spectra range characteristic for hydrogen bonding is shown in Figure 3B. Adsorption bands at 3680–3750 cm^−1^ were attributed to surface hydroxyl groups (Figure 3B, curves: c,d).

**Nanocomposite materials characterisation:** Thermal analysis (TG/DSC) of the fabricated nanocomposite materials revealed that glass transition temperature (T_g_) and malting temperature (T_m_) at DSC curves and thermal degradation temperature (T_deg_) in thermogravimeter analyses (TG) shifted towards higher values of PLDLA/hss-SiO_2_ in relation to the pure polymer.

In the results shown in Table 3, glass transition temperature Tg occurred around 50–60 °C for all samples. Melting occurred with endothermic peaks at the temperature Tm around 160–170 °C for the nanocomposite materials samples. The degradation temperature of the PLDLA was 356.7 °C and it shifted to 350.3 °C and 348.2 °C for the composite containing hss-SiO_2_ and lss-SiO_2_, respectively. These results show that the crystallinity of the material was higher when the nano-filler was hss-SiO_2_. The probable cause of this phenomenon is the higher specific surface area (SSA) (BET, shown in Table 2 in the manuscript) of the hss-SiO_2_ nanoparticles (almost 10× higher than SSA of the lss-SiO_2_) and the stronger interaction between the polymer chain and particle, resulting in increased crystallinity. The difference between the thermal degradation temperature of both nanocomposite materials was not significant, which was probably caused by the similar particle size and interaction level of both silica powders. Nevertheless, it was a proof of interaction between silica particles and the polymer chains [33].

**Figure 4:**
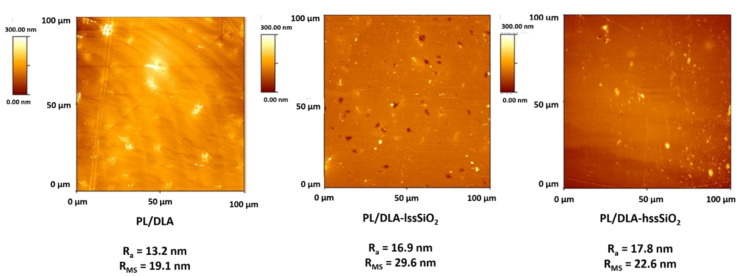
Topography of the nanocomposite materials and the polymer foil (reference sample) with characteristic parameters R_a_ and RMS.

In the case of composites, such properties can be altered by the addition of a proper type and amount of a filler. The presence of 0.5 w% of the nano-fillers in the polymer matrix influenced the roughness of the composites surface as well as it changed its physicochemical parameters. The highest mean roughness profile (RMS) was observed in the case of the composite containing lss-SiO_2_; it was 29.6 nm, compared to 22.6 nm for the composite containing hss-SiO_2_ and 19.1 nm for the pure polymer. The roughness increase was caused by the presence of surface pores that were revealed by AFM observations (Figure 4). Such surface pores did not exist in the case of the pure polymer and composite containing hss-SiO_2_. This was probably an effect of lss-SiO_2_ particles agglomeration. The solvent wetted the nanoparticles and evaporated faster from their surface than from bulk polymer, which caused the creation of pin holes on the nanocomposite surface. The results of roughness and thermal analysis of the material based on lss-SiO_2_ proved inhomogeneous dispersion of the nanofiller in the polymer matrix.

**Figure 5:**
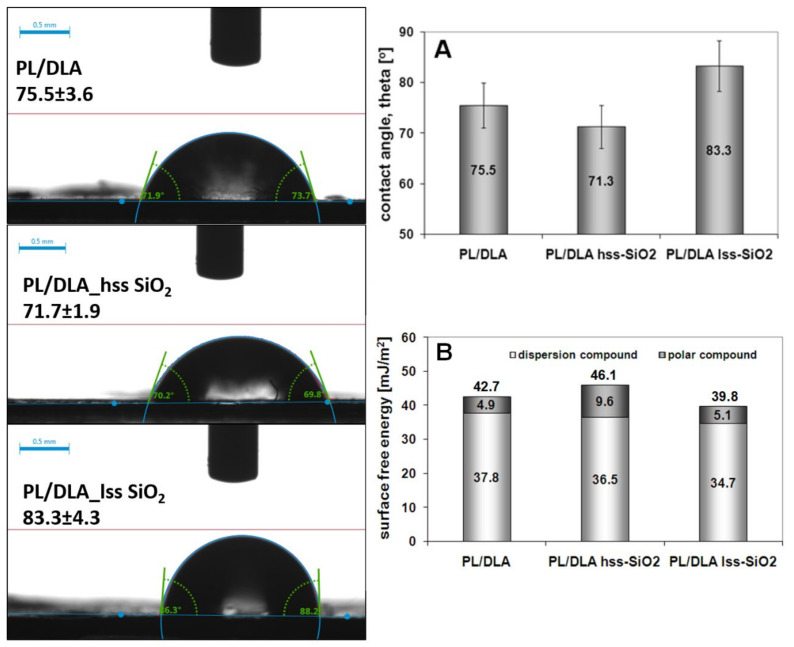
Results of contact wetting angle of the pure polymer and the nanocomposites materials (**A**) and contact angle images of the samples. Surface energy of the pure polymer and the composites (**B**).

While the addition of the silica nanopowders to the polymer matrix causes a slight change in the wettability of the nanocomposite film (Figure 5A), the value of the surface free energy is changed (Figure 5B). The addition of lss-SiO_2_ causes a decrease in the total surface free energy (about 40 mJ/m^2^), while the introduction of hss-SiO_2_ into the polymer matrix causes an increase in the total surface free energy (46 mJ/m^2^). The decrease in wettability for PLDLA/hss-SiO_2_ and the increase in surface energy may be a synergistic effect related to the surface topography of the nanocomposite [45]. The obtained results may implicate the cellular response to the material in the in vitro tests with cells.

**Figure 6:**
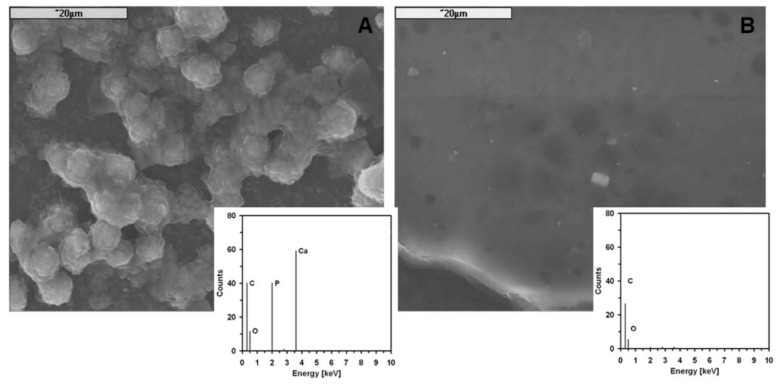
SEM microphotographs of the surface of composites containing 0.5 wt.% of: hss-SiO_2_ (**A**) and lss-SiO_2_ (**B**) and EDS analysis of the surface of the composite after SBF/3 days/ 37 °C.

Silica particles are known for their biological activity. After the incubation test (SBF/3 days/37 °C), the surface of PL/DLA hss-SiO_2_ composite was covered with characteristic apatite structures (Figure 6A), and EDS microanalysis of those structures indicated the presence of Ca and P ions characteristic for apatite. Such structures were not observed in the case of material containing lss-SiO_2_ (Figure 6B).

**Figure 7:**
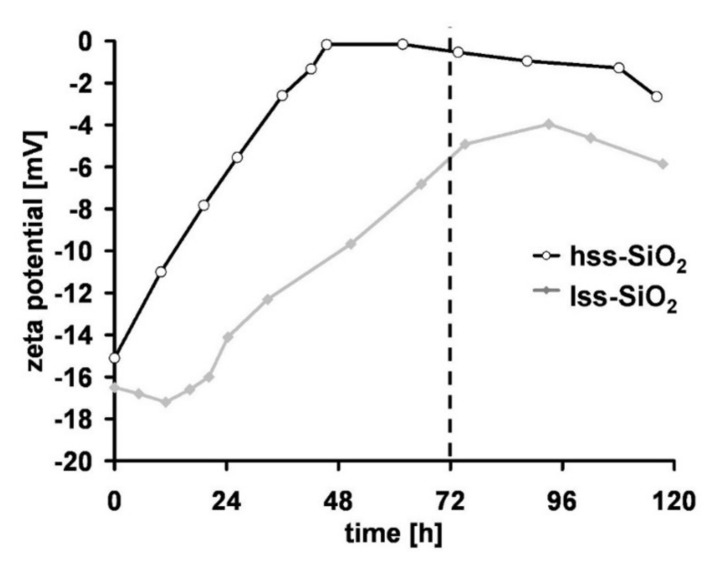
Changes of zeta potential of the silica powders vs. time (SBF, 37 °C).

Both silica powders had similar zeta potential at the beginning of the incubation in simulated body fluid (SBF). During the incubation time, zeta potential of lss-SiO_2_ was increasing until 96 h and then decreased (Figure 7). Changes of zeta potential of hss-SiO_2_ during the incubation had different characteristics; until 48 h, the potential increased to 0 mV and remained almost constant. The most important was zeta potential value after 72 h (3 days) of incubation because SEM microphotographs of the materials surface after this time revealed, in the case of the nanocomposite containing hss-SiO_2_, characteristic apatite structures. This experiment showed that nanosilica is bioactive when the potential zeta value is close to 0 mV [37]. High surface area did not influence the bioactivity of the nanosilica, but the most important feature was electric charge present on the silica nanoparticles surface.

**Figure 8:**
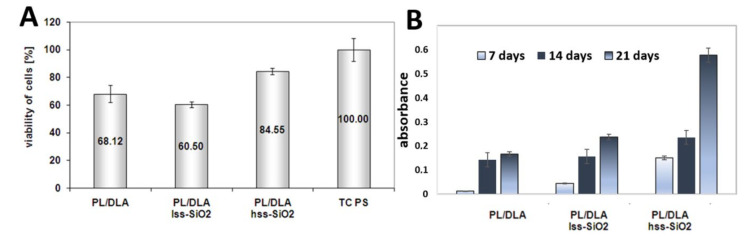
Viability of mesenchymal stem cells contacted with the nanocomposite membrane and the pure polymer membrane (**A**). TC PS was used as the reference sample. Alkaline phosphatase activity on PLDLA, PLDLA/lss-SiO_2_ and PLDLA/hss-SiO_2_ using MSCs on day 7, 14, and 21 (**B**).

**Biological studies of nanocomposite materials:** Standard test of cells viability (MTS) showed high activity of mitochondrial enzyme, which certifies biocompatibility of the material (Figure 8). Results of the viability of mesenchymal stem cells that contacted with the active surface of the nanocomposite PL/DLA showed that the material modified with hss-SiO_2_ was a better support for the bone cells, which could proliferate. This nanocomposite was also characterized by bioactivity futures, which can be an important parameter in the differentiation of osteoblast cells [46]. The MSCs were grown on PLDLA/hss-SiO_2_ nanocomposites and showed significantly higher (*p* < 0.05) proliferation levels, compared to those grown on PLDLA foil and PLDLA/lss-SiO_2_ nanocomposite, due to the presence of a domain on the surface, which decreased the hydrophobicity of the nanocomposite for adhesion of cells (Figure 8A). It is known that alkaline phosphatase (ALP) is a primary phenotypic indicator secreted by osteoblasts. The intensity of ALP secretion indicates the early osteogenesis stage. Level assessment of ALP in the fabricated PLDLA, PLDLA/lss-SiO_2_ and PLDLA/hss-SiO_2_ foils will help to validate differentiation of MSCs towards anosteogenic lineage. As observed in Figure 8B, PLDLA/hss-SiO_2_ nanocomposite foil shows a significantly increased (*p* < 0.05) level of ALP expression when compared to PLDLA and PLDLA/lss-SiO_2_ after 14 and 21 days.

**Figure 9:**
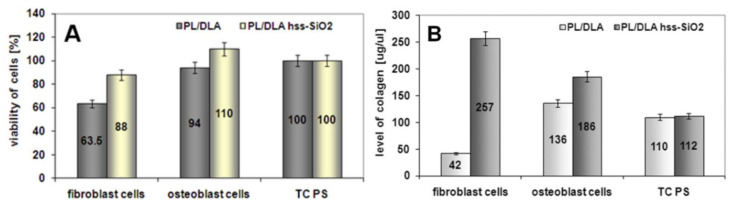
The viability of osteoblast-like cells by hFOB 1.19 cells and fibroblasts HS-5 cells contacted with the nanocomposite materials and the pure polymer foil (**A**). Level of type-I collagen produced by osteoblast and fibroblast cells (**B**). TC PS was used as a reference sample.

Comparing two kinds of nanocomposites, the more promising material is polylactide modified with hss-SiO_2_ nanoparticles. For this reason, a next biological experiment was conducted. Nanocomposite PL/DLA foil containing hss-SiO_2_ particles were contacted with osteoblast-like cells (hFOB 1.19) and fibroblast cells (HS-5). After 3 days of the incubation, viability of both types of cells was higher in the case of the nanocomposite than the cells contacted with the pure polylactide. After 7 days of the incubation, metabolic activity of these cells was tested by determination of the concentration of type I collagen. This peptide acted in the experiment as an indicator of the metabolic activity. The level of the collagen produced by hFOB 1.19 cells and HS-5 cells was higher in the case of the nanocomposite foils than on the pure polymer (Figure 9). These results confirmed the metabolic activity of osteoblasts on the nanocomposite containing hss-SiO_2_. It was shown that the nanocomposite surface topography created by the silica nanoparticles was more attractive for fibroblast cells than for osteoblast cells. Production of the collagen assigned to fibroblasts cells was 1.4 times higher than the production of the osteoblast cells.

## 4. Discussion

The possibility of nucleation of apatite on micrometric silica particles and silica gel was tested in laboratories in the 1990s [47,48]. It was shown then that the chemical activity of micrometric particles strongly depends less on the surface chemistry of the microparticle (specifically on the method of its preparation) than on its microstructure (presence of micropores) [47]. It was then suggested that apatite formation in SBF occurs as a result of high concentration of surface silanol groups, which may constitute a fordable site for apatite nucleation [49]. Other researchers pointed to the possibility of apatite nucleation through the formation of silanol–phosphate complexes, as it occurs during the formation of a gel layer in bioglass [50]. More importantly, observations made on properly prepared micrometric silica particles can be directly translated into the behaviour of a group of bioactive glasses [50,51]. The phenomena known from the nature of micrometric SiO_2_ particles do not always translate into the behaviour of chemically identical but nanometric silica particles. The analysis of two types of nanometric SiO_2_ particles differing in their specific surface area showed a synergic effect of surface chemistry and microstructure of the nanoparticle on the maintenance of biological activity (Kokubo test, SBF [52]) both for the particles and for the polymer nanocomposites with their participation. The differences in the specific surface area result from the presence of micropores (i.e., pores of diameter smaller than 2 nm) in the hss-SiO_2_ nanoparticles. Cho and al. suggested that mesopores in the range from 2 to 200 nm present on the material’s surface did not influence nucleation of an apatite layer. On the other hand, micropores smaller than 2 nm induced the formation of apatite, playing a role of the center of apatite nucleation (similar to a defect in traditional materials) [47].

Nanometric silica with a high specific surface area is additionally characterized by chemical groups typical for the nanoparticles (e.g., for SiO_2,_ the groups are Si-O in air and Si-OH in water conditions) are exposed on the surface of the nanoparticles (FTIR study, Figure 3). Some Researchers suggested that these bands could be attributed to geminal hydroxyls Si-(OH)_2_ [43,44]. These bands were much more intensive in hss-SiO_2_ than in lss-SiO_2_. Weak OH bands in the 3680–3750 cm^−1^ range can only be captured when using a special powder spectroscopic examination technique such as DRIFTs; other spectroscopic methods are unable to capture such nuances. In this case, it means that the large surface area of hss-SiO_2_ (BET, shown in Table 2) adsorbs water from the air very quickly, which causes the appearance of chemically active OH groups. Probably, the hydroxyl groups are able to generate the second-row interactions between the polymer chain and the hss-SiO_2_ nanoparticle, hence its bioactivity (the Zeta potential indicating bioactivity of the hss-SiO_2_ after 48 h of the incubation in SBF—Figure 7) and higher viability and metabolic activity of mesenchymal cells (Figure 8A,B). Such properties were not present in the lss-SiO_2_ nanoparticle, which results in lower crystallinity comparable to the pristine polymer (PLDLA), as well as a lack of bioactivity (no nucleation of apatite on the surface, Figure 6B) and lower biological activity is visible in the viability and metabolic activity of the mesenchymal cells (Figure 8A,B). In the case of polymer–ceramic materials, the formation of a nanocomposite is accompanied by various phenomena, which are not present in the case of conventional composite materials. They are caused by the interaction between polymer chains and fine particles introduced into a polymer matrix.

In the case of polymer–ceramic materials, the formation of a nanocomposite is accompanied by various phenomena, which are not present in the case of conventional composite materials. They are caused by the interaction between polymer chains and fine particles introduced into a polymer matrix. Introduction of a filler with numerous active chemical groups into the polymer matrix affects the degree of short-range order (structure) and also its microstructure. SiO_2_ nanoparticles with smaller specific surface area show stronger tendency for agglomeration, which manifests in a change of surface topography (RMS) of the nanocomposite (surface pores in PLDLA/lss-SiO_2_) and decrease of its crystallinity. No changes in the topography of the nanocomposite but also an increase in its crystallinity and a shift of the melting temperature of the material towards lower values in comparison with the pure polymer confirms better compatibility of hss-SiO_2_ nanoparticles with PLDLA matrix [33,53,54]. The high dispersion degree of hss-SiO_2_ nanoparticles induces physicochemical changes in the surface—an increase in hydrophobicity while maintaining the ratio between the polar and dispersion component at the level of the pure polymer. The literature reports examples of materials with similar behaviour, which are caused by the reorganization of the polymer chain through interaction with the nanoparticles [55,56]. The drying stage of the nanocomposite may be responsible for changes in surface properties; incorrect conditions lead to the process of secondary agglomeration [57]. It seems that such a negative phenomenon of the secondary agglomeration occurred in the case of PLDLA/lss-SiO_2_ nanocomposite; a change in wettability, increase in the dispersion component, and at the same time a change in topography confirmed lower compatibility of the filler to the polymer matrix. The usefulness of prepared PLDLA/SiO_2_ nanocomposites as potential bone substitute materials was confirmed by a bioactivity test in which apatite forms were observed on the PLDLA/hss-SiO_2_ surface (Figure 6). Based on the literature data, it is possible to predict the chemical activity of e.g., bio-glass by testing the zeta potential in the simulated body fluid. The authors of these papers define the kinetics of the appearance of the Ca-P layer in in vitro conditions and relate it to the bioactive potential in in vivo conditions [58,59].

Using a similar methodology to the studies on the suitability of nanoparticles and nanocomposites with their presence, the time needed to change the zeta potential value from a negative one (typical of the SiO_2_ gel, Si-OH) to a slightly positive one was determined. In this time, calcium and then phosphate ions responsible for the Ca-P layer and cauliflower-like apatite forms are accumulated [34,37,60]. Nanometric silica with a highly developed surface area is bioactive when the zeta potential is close to 0 mV. It reaches this value after 72 h, which makes it as chemically active as the Hench bioglass.

Probably part of the silica particles was exposed on the composite surface and their physicochemical properties strongly influenced the bioactivity of the material. It seems that the key factor was the specific surface area of the silica powders. The hss-SiO_2_ had higher specific surface area, which was mainly a result of the presence of micropores. It enables to assume that there was more O-Si-O (silane) groups, which were able to form bonds between the material and surroundings, which in turn facilitated crystallisation of apatite [37,61].

Surface properties of a biomaterial are the key factor responsible for their biological response [62,63]. Cell–materials interaction strongly depends on the material’s surface properties [28]. Osteoblast cells are very sensitive to many properties of surface such as: roughness, wettability, surface free energy, and its chemical structure [64]. The previous results (structure, wettability, surface free energy, and morphology) presented in this paper showed that nanocomposite materials based on polylactide were characterized by different microstructures and domain-like surfaces in which some areas were modified by the nanofillers particles that interacted with the polymer matrix (PLDLA/lss-SiO_2_). Biological studies of the nanocomposites in contact with mesenchymal stem cells (MSCs) derived from bone marrow provided promising results. Such cells are more sensitive investigation tools than usually applied cells from the continuous lines [65,66,67]. The ALP activity was significantly increased in nanocomposites foil PLDLA/hss-SiO_2_ when compared to other nanocomposites foils and polymer-based materials on day 21. Researchers reported that silica-coated nanoparticles stimulate the osteogenic differentiation of bone marrow MSCs in vitro concomitant with the upregulation of ALP activity [68,69]. Nanosilica may induce in vitro osteogenic differentiation of precursor cells, as well as improve in vitro osteogenic formation.

## 5. Conclusions

Results indicate that nanometric dispersion of the nano-fillers (hss-SiO_2_, lss-SiO_2_) in a resorbable matrix of poly(L-lactide-co-D,L-lactide) is a promising route for the preparation of bioactive nanocomposite implants destined for applications in bone surgery. However, properties of silica nanopraticles such as specific surface area, size, zeta potential, and amount of chemical active groups on the surface strongly influence the biological response of cells to the nano-silica filled materials. They include:

- the particles of the high specific surface silica (hss-SiO_2_) in polymer matrix (PLDLA) significantly enhanced the formation of apatite-like structures at the surface during a bioactivity test (in vitro conditions); this feature can be monitored by zeta potential in SBF;
- the nanofillers such as hss-SiO_2_ significantly influence physicochemical properties such as wetting contact angle and surface free energy of polymer matrix, and slightly influence the crystallinity of nanocomposite materials (PLDLA/hss-SiO_2_). These parameters may be relevant to the biological response from both the mesenchymal and somatic cells;
- the results of biological investigations, realised with the use of mesenchymal stem cells, show that the bioactive nanocomposite where hss-SiO_2_ was used as a filler may stimulate differentiation of mesenchymal cells into osteoblasts—resulting in higher proliferation stage of cells in in vitro conditions and higher alkaline phosphatase activity. Somatic cells: fibroblast and osteoblast contacted with nanocomposite with hss-SiO_2_ confirm that this material is much more suitable for promotion of cells’ proliferation than PLDLA/lss-SiO_2_.

The results showed also that the bioactivity of nanocomposite materials can be anticipated by the zeta potential of nanoadditives. This can also be useful in the fabrication of nanocomposites with homogenous dispersion of nanofillers in a polymer matrix. Thorough characterization of nanofillers belonging to the same group of materials i.e., ceramic nanoparticles, may help to design the nanocomposite 3D scaffolds.

## Figures and Tables

**Table 1:** Chemical composition of simulated body fluid (SBF) and body plasma (pH = 7.4) [25,40].

Fluid	Concentration of Ion (mM)							
	Cl^−^	HCO_3_^−^	HPO_4_^2−^	SO_4_^2−^	Na^+^	K^+^	Mg^2+^	Ca^2+^
**Body Plasma**	103.0	27.0	1.0	0.5	142.0	5.0	1.5	2.5
**Simulated Body Fluid, SBF**	148.8	4.2	1.0	0.5	142.0	5.0	1.5	2.5

**Table 2:** Characteristics of the silica nanoparticles used in experiments.

Nanoparticles Characteristics	hss-SiO_2_	lss-SiO_2_
Modal particle diameter, DLS (nm)	220	142
Mean particle size, TEM (nm)	60	50
Specific surface area, BET (m^2^/g)	582.8	65.8

**Table 3:** Thermal properties of the nanocomposite foils and the pure polymer.

Material	T_g_, °C	T_m_, °C	T_deg_, °C	λ, %
PL/DLA	57.3	162.5	356.7	36.5
PL/DLA hss-SiO_2_	59.8	168.2	350.3	40.2
PL/DLA lss-SiO_2_	57.2	161.8	348.2	37.6

## Data Availability

Not applicable.
